# Risk prediction for recurrent pregnancy loss based on routine inspections in the first trimester of pregnancy, a retrospective study in China

**DOI:** 10.3389/fmed.2025.1476722

**Published:** 2025-04-09

**Authors:** Jinming Wang, Dan Li, Yang Yeung, Zhenglong Guo, Hongke Huang, Li Wang, Weili Shi, Jianmei Huang, Wenke Yang, Yanxin Ren, Shixiu Liao, Yibin Hao

**Affiliations:** ^1^Medical Genetics Institute of Henan Province, Henan Provincial People’s Hospital, Zhengzhou University People’s Hospital, Zhengzhou, China; ^2^Institute of Information Technology, PLA Strategic Support Force Information Engineering University, Zhengzhou, China; ^3^Medical Laboratory Technology Department, Huanghe Science and Technology College, Zhengzhou, China

**Keywords:** recurrent pregnancy loss, risk prediction, routine inspections, the first trimester of pregnancy, logistic regression analysis, retrospective study

## Abstract

**Background:**

Recurrent pregnancy loss (RPL) is one of the most common pregnancy complications in obstetrics and gynecology, and the incidence rate of RPL is about 2%. To establish a risk prediction model for recurrent pregnancy loss based on routine inspections in the first trimester of pregnancy.

**Materials and methods:**

A total of 3,010 women at Henan Provincial People’s Hospital between January 2019 and December 2023. 810 women at Shangqiu Maternal and Child Health Hospital between January 2021 and April 2024. There were 523 women in the training set, 282 women in the testing set, and 229 women in the external validation set. Twelve routine inspections in the first trimester of pregnancy (4 ~ 12 weeks) were collected including thyroid-stimulating hormone (TSH), free triiodothyronine (FT3), free thyroxine thyroid (FT4), thyroxine (TT4), total triiodothyronine (TT3), peroxidase antibody (TPO-Ab), thyroid globulin antibody (TG-Ab), 25-hydroxyvitamin D (25-(OH) D), ferritin (Ferr), Homocysteine (Hcy), vitamin B12 (VitB12), folic acid (FA). Logistic regression analysis was used to establish a risk prediction model based on training set. Receiver operating characteristic (ROC) curve and decision curve analysis (DCA) were employed to evaluate the performance of prediction model on testing set and external validation set.

**Results:**

Chi-square test results for each single characteristic indicated that, TPO-Ab (*p* = 0.005), TG-Ab (*p* < 0.001), 25-(OH) D (*p* < 0.001), Hcy (*p* < 0.001) and FA (*p* < 0.001) were closely related to RPL. The prediction accuracy of the logistic regression model on the testing set was 71.28%, and area under ROC curve was 0.766. The prediction accuracy of the model on external validation set was 69.87%, and area under ROC curve was 0.759. Calibration curve and DCA curves of testing set and external validation set indicated that the model had good clinical value.

**Conclusion:**

TPO-Ab, TG-Ab, 25-(OH) D, Hcy and FA may be closely related to the occurrence and development of RPL. The model only requires routine inspections in the first trimester of pregnancy to effectively indicate high-risk groups of RPL before the first miscarriage, making it convenient for clinical application and implementation.

## Introduction

Recurrent pregnancy loss (RPL) is one of the most common pregnancy complications in obstetrics and gynecology, and the incidence rate of RPL is about 2% (1–3). Patients with RPL usually have experienced multiple miscarriages before diagnosis, which is not only a serious threat to the physical and mental health of patients, but also a heavy economic burden to patients and their families (1). Due to the complex and diverse etiology of RPL, as well as the lack of specific clinical manifestations in patients before pregnancy loss occurs, risk prediction and early intervention of RPL have become important issues that urgently need to be addressed in the field of reproductive health (1, 4).

Epidemiological investigation showed the number of expected abortions was the main risk factors for pregnancy loss (2, 5). However, a systematic meta-analysis showed that there were no differences in the incidence of uterine abnormalities, chromosomal abnormalities, thrombotic diseases, and thyroid diseases between patients with 2 and ≥ 3 failed pregnancies, indicating statistical differences between RPL patients and the normal pregnancy women (6). These differences may already exist before the first pregnancy loss. If these differences can be detected earlier, it is of great significance for identifying high-risk populations for RPL and improving prognosis (3, 5).

The causes of RPL mainly include chromosomal or genetic abnormalities, anatomical abnormalities (including congenital and acquired), autoimmune diseases, pre-thrombotic states, endocrine factors, infectious factors, male factors, and environmental psychological factors (2, 6). It should be pointed out that the specific causes and pathogenesis of about 40% of RPL are still unknown (7). In clinical diagnosis and treatment, the screening indicators of RPL involve multiple personalized examination items such as reproductive tract anatomy, chromosome karyotype, autoimmune antibodies and thromboela-stogram, which are often used for etiological analysis after the diagnosis of RPL, but not suitable for evaluating the risk of RPL before the miscarriage occurs (7). It is very difficult to conduct effective early intervention before the diagnosis of RPL.

Thyroid function indicators, 25-(OH) D, ferritin, homocysteine, vitamin B12 and folic acid are relatively routine inspections for women in the first trimester of pregnancy (7–11). Previous studies have shown that some of these factors may be related to RPL, but the clinical reliability and accuracy of predicting the risk of RPL based on a single indicator are difficult to guarantee (7, 10). In recent years, artificial intelligence and machine learning have been successfully applied in disease diagnosis, disease development prediction, disease risk factor identification, and new drug development in the medical field (11–13). Logistic regression is a classic machine learning method, which can statistically analyze the impact of multiple variables on diseases and is more helpful for clinical practical applications (13–15).

The main purpose of this study is to construct a risk prediction model for RPL based on routine inspections in the first trimester of pregnancy, in order to provide clinical physicians with indications of whether the patients are at high risk before the miscarriage occurs. This study has important value for early intervention and improving prognosis in patients with RPL. To our knowledge, this study is the first time to assess the risks of RPL based on routine inspections in the first trimester of pregnancy using logistic regression.

## Materials and methods

### Study design

**Figure 1:**
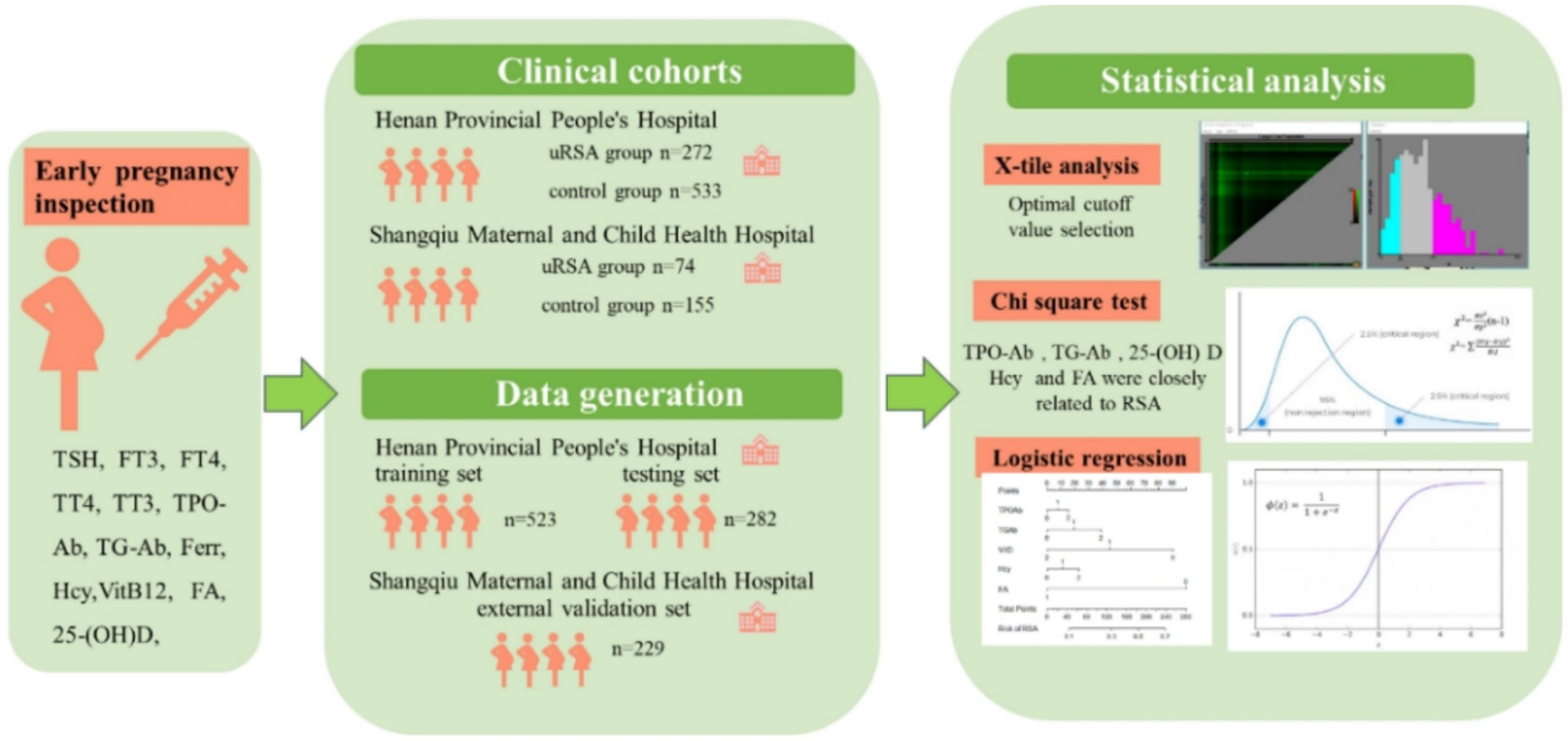
Schematic diagram of the study design.

This was a retrospective study at Henan Provincial People’s Hospital, Prenatal Diagnosis Center and Shangqiu Maternal and Child Health Hospital. The study protocol was approved by the Ethics Committees of Henan Provincial People’s Hospital and Shangqiu Maternal and Child Health Hospital with a waiver for informed consent. The research methods were carried out in accordance with relevant guidelines and regulations. 8 personal basic information and 12 inspections in the first trimester of pregnancy for each participant were collected. Participants were divided into training set, testing set and external validation set. X-tile analysis, chi-square test and logistic regression were used for statistical analysis. The overall workflow of this study and detailed participant recruitment information for each analysis are shown in Figure 1.

### Participants

This study involved a total of 3,010 women divided in RPL group (1,156 women) and control group (1854 women) at Henan Provincial People’s Hospital between January 2019 and December 2023. RPL was defined as 2 or more consecutive pregnancy losses before 24 weeks’ gestation (1). The inclusion criterion for control group was the occurrence of at least one normal delivery with no history of pregnancy loss. When collecting participant information, we recorded the number of pregnancies, number of losses and number of live births to distinguish between the RPL group and the control group.

In this study, we focused on the relationship between 12 inspections in the first trimester of pregnancy (4 ~ 12 weeks) and RPL. The inspections included thyroid-stimulating hormone (TSH), free triiodothyronine (FT3), free thyroxine thyroid (FT4), thyroxine (TT4), total triiodothyronine (TT3), peroxidase antibody (TPO-Ab), thyroid globulin antibody [(TG-Ab), 25-hydroxyvitamin D (25-(OH) D], ferritin (Ferr), homocysteine (Hcy), vitamin B12 (VitB12), folic acid (FA). There are two reasons for choosing the above indicators. Firstly, these indicators are routine inspections, so almost all pregnant women undergo them. Secondly, previous studies have shown that these inspections may be correlated with the occurrence and development of RPL. Given the correlation between indicators such as homocysteine and adverse pregnancy outcomes, since 2017, the Prenatal Diagnosis Center of Henan Provincial People’s Hospital has recommended that all women in their first trimester of pregnancy undergo these 12 tests. Participants are fully informed of the risks associated with abnormalities in these indicators, and the final decision on which tests to undergo is made by them.

We retrieved the inspection records of women in RPL group before the occurrence of the first pregnancy loss through the hospital outpatient system but found 356 women without that inspection records and 379 women without enough inspection items required in this study. Similarly, we retrieved the inspection records of women in control group before the occurrence of the first normal delivery but found 418 women without that inspection records and 580 women without enough inspection items required in this study. We also recorded some baseline characteristics including age, body mass index (BMI), smoking history, drinking history, regular menstrual cycle at that time.

**Figure 2:**
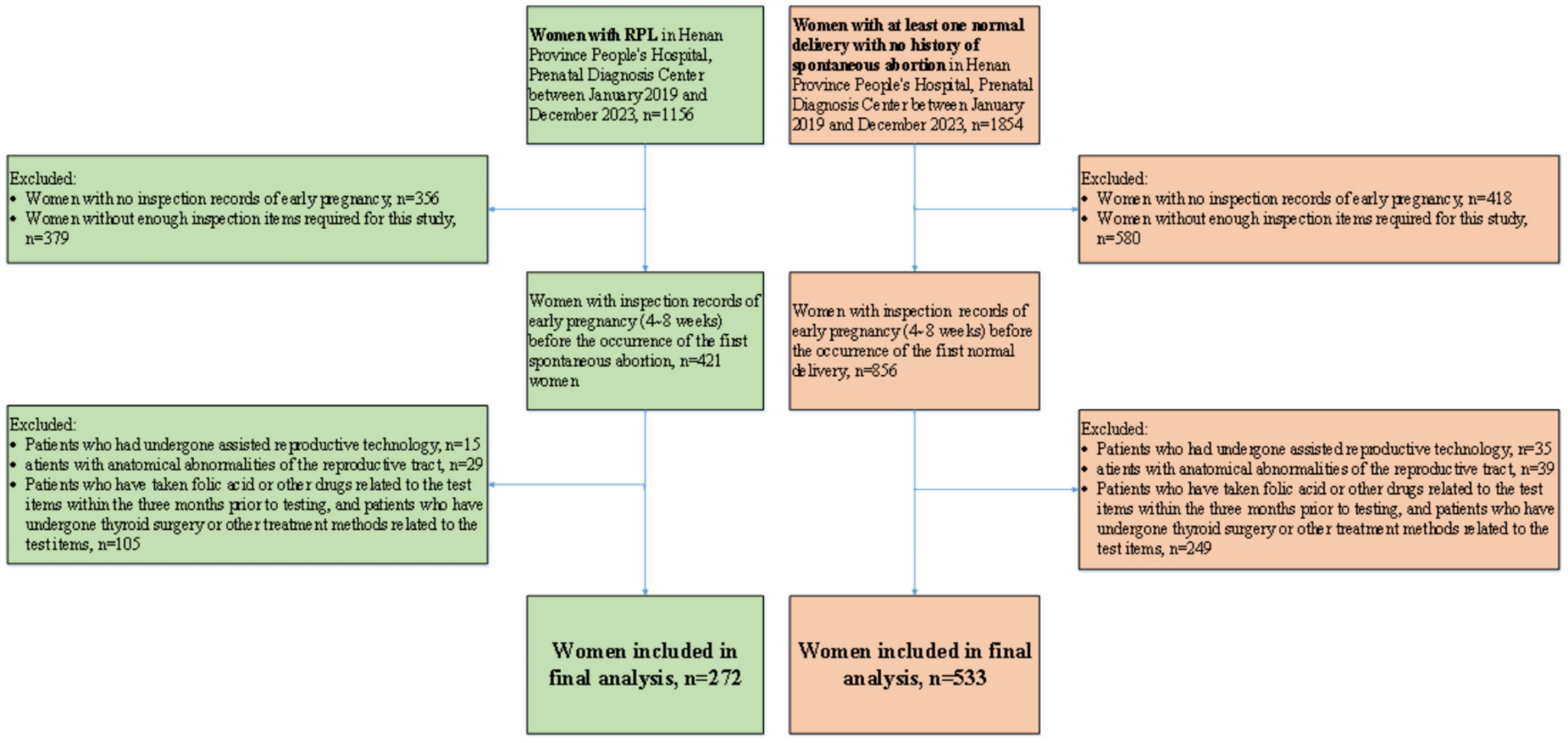
Flowchart of participants at Henan Provincial People’s Hospital.

This study aimed to screen high-risk individuals for RPL from women in the first trimester of pregnancy. The exclusion criteria include three groups of people. (1) We excluded patients who have received assisted reproductive technology, which were not within the scope of this study. (2) We excluded patients with anatomical abnormalities of the reproductive tract, which can be detected through routine ultrasound examination before or during early pregnancy. (3) We excluded patients who have taken folic acid or other drugs related to the test items within the 3 months prior to testing, and patients who have undergone thyroid surgery or other treatment methods related to the test items, because their detection results were interfered and could not display the true level. Through these exclusion criteria, 149 women were excluded from the RPL group and 323 women were excluded from the control group, respectively. Finally, 272 women in RPL group and 533 women in control group were included in the study. Flowchart of participant inclusion process is shown in Figure 2.

**Figure 3:**
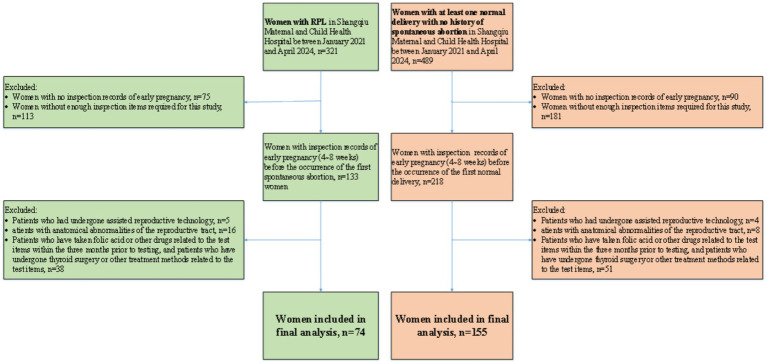
Flowchart of participants at Shangqiu Maternal and Child Health Hospital.

In order to verify the universality of the conclusions of this study, we collected 810 women who met the inclusion criteria at the Shangqiu Maternal and Child Health Hospital between January 2021 and April 2024. We also excluded women whose inspection records were missing or incomplete and strictly followed the exclusion criteria. Finally, 74 women in RPL group and 155 women in control group were included as external validation set in the study. Flowchart of participant inclusion process is shown in Figure 3. It should be noted that due to the different units selected for some inspection indicators between Shangqiu Maternal and Child Health Hospital and Henan Provincial People’s Hospital, the units were unified during the data collation process.

### Statistical analysis

In order to improve the convergence performance of the model, we selected the optimal cutoff value based on X-tile analysis and clinical experience, and converted continuous variables into categorical variables. Chi-square test was used to analyze the significance of differences in various characteristics between RPL patients and the control group, and *p* < 0.05 was considered to be statistically significant. 272 women in RPL group and 533 women in control group were randomly split into two distinct subsets: a training set with 523 women (65%) to construct the model and a testing set with 282 women (35%) to test the model. Logistic regression analysis was used to establish a risk prediction model according to training set, and receiver operating characteristic (ROC) curve, calibration curve and decision curve analysis (DCA) were employed to evaluate the performance of prediction model on testing set and external validation set. All statistical analyses were performed with the use of SPSS version 27.

## Results

### Information of participants

The participants in this study were from two hospitals. Characteristics of 805 participants from Henan Provincial People’s Hospital was used for model training and internal testing, and characteristics of 229 participants from Shangqiu Maternal and Child Health Hospital was used for external validation. Table 1 shows the analysis of differences in participants from different hospitals. There was no statistically significant difference in all characteristics (*p* > 0.05).

**Table 1:** Analysis of differences in participants from different hospitals.

Characteristics	Participants at Henan Provincial People’s hospital (*n* = 805)	Participants at Shangqiu maternal and child health hospital (*n* = 229)	*p* value
Age			0.822
<25	49(6.09%)	15(6.55%)	
25 ~ 35	678 (84.22%)	189 (82.53%)	
≥35	78 (9.69%)	25 (10.92%)	
BMI			0.156
<24	501 (62.24%)	129 (56.33%)	
24 ~ 28	270 (33.54%)	85 (37.12%)	
≥28	34 (4.22%)	15 (6.55`%)	
Smoking history*			0.355
No	753 (93.54%)	218 (95.20%)	
Yes	52 (6.46%)	11 (4.80%)	
Drinking history*			0.910
No	646 (80.25%)	183 (79.91%)	
Yes	159 (19.75%)	46 (20.09%)	
Regular menstrual cycle			0.749
Yes	740 (91.93%)	209 (91.27%)	
No	65 (8.07%)	20 (8.73%)	
Number of pregnancies			0.711
1	392 (48.70%)	116 (50.66%)	
2	319 (39.63%)	84 (36.68%)	
≥3	94 (11.67%)	29 (12.66%)	
Number of losses			0.728
0	533 (66.21%)	155 (67.69%)	
1 ~ 2	243 (30.19%)	64 (27.95%)	
≥3	29 (3.60%)	10 (4.36%)	
Number of live births			0.838
0	235 (29.19%)	65 (28.38%)	
1 ~ 2	535 (66.46%)	152 (66.38%)	
≥3	35 (4.35%)	12 (5.24%)	
TSH			0.839
<1.2 uIU/mL	185 (22.98%)	49 (21.40%)	
1.2 ~ 3.3 uIU/mL	500 (62.11%)	147 (64.19%)	
≥3.3 uIU/mL	120 (14.91%)	33 (14.41%)	
FT3			0.885
<3.8 pmol/mL	79 (9.81%)	25 (10.92%)	
3.8 ~ 4.2 pmol/mL	201 (24.97%)	57 (24.89%)	
≥4.2 pmol/mL	525 (65.22%)	147 (64.19%)	
FT4			0.904
<12.6 pmol/mL	222 (27.58%)	63 (27.51%)	
12.6 ~ 13.8 pmol/mL	245 (30.43%)	73 (31.88%)	
≥13.8 pmol/mL	338 (41.99%)	93 (40.61%)	
TT4			0.591
<102 nmol/L	367 (45.59%)	109 (46.29%)	
≥102 nmol/L	438 (54.41%)	120 (53.71%)	
TT3			0.873
<1.6 nmol/L	558 (69.32%)	160 (69.87%)	
≥1.6 nmol/L	247 (30.68%)	69 (30.13%)	
TPO-Ab			0.912
<1.5 IU/mL	415 (51.55%)	117 (51.09%)	
1.5 ~ 24.5 IU/mL	293 (36.40%)	82 (35.81%)	
≥24.5 IU/mL	97 (12.05%)	30 (13.10%)	
TG-Ab			0.738
<1.2 IU/mL	322 (40.00%)	95 (41.49%)	
1.2 ~ 28 IU/mL	365 (45.34%)	105 (45.85%)	
≥28 IU/mL	118 (14.66%)	29 (12.66%)	
VitD			0.343
<7.8 ng/mL	178 (22.11%)	55 (24.02%)	
7.8 ~ 10.3 ng/mL	190 (23.60%)	62 (27.07%)	
≥10.3 ng/mL	437 (54.29%)	112 (48.91%)	
Ferr			0.911
<32 ng/mL	376 (46.71%)	106 (46.29%)	
≥32 ng/mL	429 (53.29%)	123 (53.71%)	
Hcy			0.285
<8.9 μmol/mL	491 (60.99%)	127 (55.46%)	
8.9 ~ 11.2 μmol /mL	167 (20.75%)	57 (24.89%)	
≥11.2 μmol /mL	147 (18.26%)	45 (19.65%)	
VitB12			0.166
<330 pg./mL	166 (20.62%)	57 (24.89%)	
≥330 pg./mL	639 (79.38%)	172 (75.11%)	
FA			0.849
<5.70 ng/mL	589 (73.17%)	169 (73.80%)	
≥12.1 ng/mL	216 (26.83%)	60 (26.20%)	

*Smoking history refers to smoking at least one cigarette per day for at least six months. Drinking history refers to drinking alcohol at least once a week and consuming ethanol more than 20 g.

### Baseline characteristics

Table 2 shows the baseline characteristics of 272 women in RPL group and 533 women in control group. As for the baseline characteristics including age, BMI, smoking history, drinking history and regular menstrual cycle, no statistically significant differences were found between the two groups (*p* > 0.05).

**Table 2:** Baseline characteristics.

Characteristics	Control group (*n* = 533)	RPL group (*n* = 272)	*p* value
Age			0.340
<25	34 (6.38%)	15 (5.51%)	
25 ~ 35	453 (84.99%)	225 (82.72%)	
≥35	46 (8.63%)	32 (11.76%)	
BMI			0.402
<24	325 (60.98%)	176 (64.71%)	
24 ~ 28	187 (35.08%)	83 (30.51%)	
≥28	21 (3.94%)	13 (4.78%)	
Smoking history*			0.863
No	499 (93.62%)	255 (93.75%)	
Yes	34 (6.38%)	17 (6.25%)	
Drinking history*			0.540
No	431 (80.86%)	215 (79.04%)	
Yes	102 (19.14%)	57 (20.96%)	
Regular menstrual cycle			0.992
Yes	490 (91.93%)	250 (91.91%)	
No	43 (8.07%)	22 (8.09%)	

*Smoking history refers to smoking at least one cigarette per day for at least six months. Drinking history refers to drinking alcohol at least once a week and consuming ethanol more than 20 g.

### Significance analysis

To determine the risk factors related to RPL, chi-square test results for each single characteristic between RPL group and control group are presented in Table 3. Only TPO-Ab (*p* = 0.005), TG-Ab (*p* < 0.001), 25-(OH) D (*p* < 0.001), Hcy (*p* < 0.001) and FA (*p* = 0.001) were closely related to RPL among 12 inspections in the first trimester of pregnancy.

**Table 3:** Chi-square test for each single factor.

Characteristics	Control group (*n* = 533)	RPL group (*n* = 272)	*χ^2^*	*p* value
TSH				
<1.2 uIU/mL	122 (22.88%)	63 (23.16%)	0.308	0.857
1.2 ~ 3.3 uIU/mL	334 (62.66%)	166 (61.03%)		
≥3.3 uIU/mL	77 (14.46%)	43 (15.81%)		
FT3				
<3.8 pmol/mL	46 (8.63%)	33 (12.13%)	3.030	0.220
3.8 ~ 4.2 pmol/mL	139 (26.08%)	62 (22.79%)		
≥4.2 pmol/mL	348 (65.29%)	177 (65.07%)		
FT4				
<12.6 pmol/mL	148 (27.77%)	74 (27,21%)	0.074	0.964
12.6 ~ 13.8 pmol/mL	163 (30.58%)	82 (30.15%)		
≥13.8 pmol/mL	222 (41.65%)	116 (42.64%)		
TT4				
<102 nmol/L	246 (46.15%)	121 (44.49%)	0.202	0.653
≥102 nmol/L	287 (53.85%)	151 (55.51%)		
TT3				
<1.6 nmol/L	369 (69.23%)	189 (69.49%)	0.005	0.141
≥1.6 nmol/L	164 (30.77%)	83 (30.51%)		
TPO-Ab				
<1.5 IU/mL	295 (55.35%)	120 (44.12%)	10.509	0.005
1.5 ~ 24.5 IU/mL	174 (32.65%)	119 (43.75%)		
≥24.5 IU/mL	64 (12.00%)	33 (12.13%)		
TG-Ab				
<1.2 IU/mL	240 (45.03%)	82 (30.15%)	19.376	<0.001
1.2 ~ 28 IU/mL	214 (40.15%)	151 (55.51%)		
≥28 IU/mL	79 (14.82%)	39 (14.34%)		
VitD				
<7.8 ng/mL	67 (12.57%)	111 (40.81%)	91.196	<0.001
7.8 ~ 10.3 ng/mL	126 (23.64%)	64 (23.53%)		
≥10.3 ng/mL	340 (63.79%)	97 (35.66%)		
Ferr				
<32 ng/mL	256 (48.03%)	120 (44.12%)	1.107	0.293
≥32 ng/mL	277 (51.97%)	152 (55.88%)		
Hcy				
<8.9 μmol/mL	364 (68.29%)	127 (46.69%)	35.525	<0.001
8.9 ~ 11.2 μmol /mL	88 (16.51%)	79 (29.04%)		
≥11.2 μmol /mL	81 (15.20%)	66 (24.27%)		
VitB12				
<330 pg./mL	114 (21.39%)	52 (19.12%)	0.567	0.451
≥330 pg./mL	419 (78.61%)	220 (80.88%)		
FA				
<5.70 ng/mL	339 (63.60%)	250 (91.91%)	73.517	<0.001
≥12.1 ng/mL	194 (36.40%)	22 (8.09%)		

### Prediction model using logistic regression

**Figure 4:**
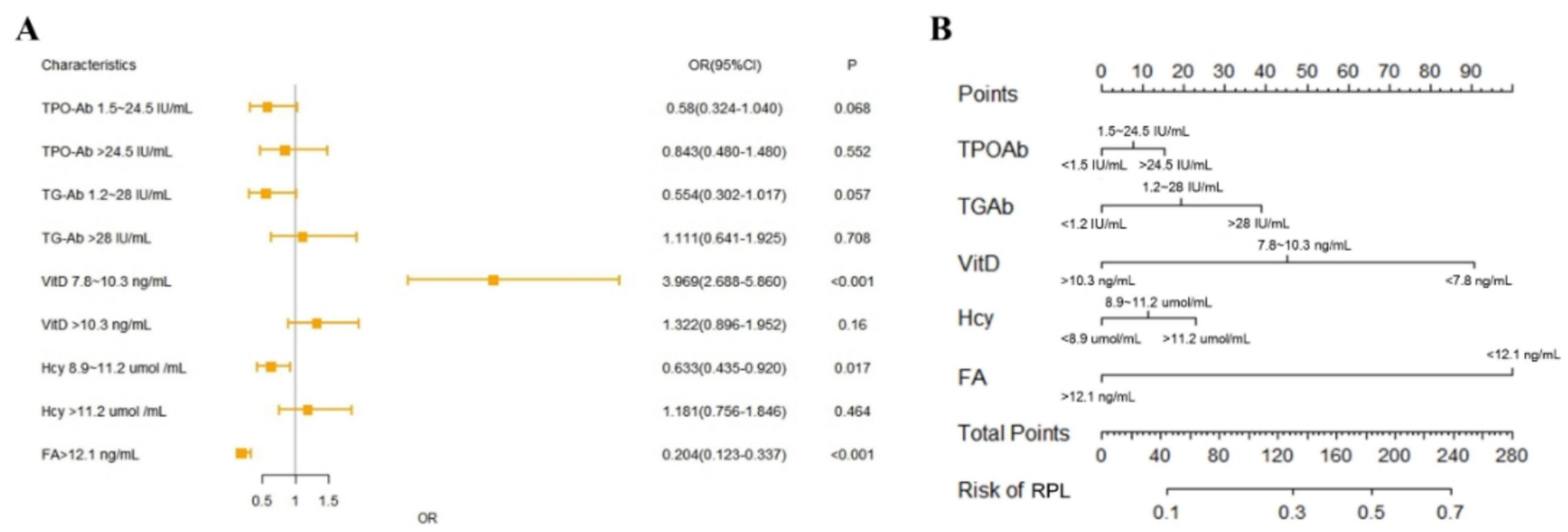
Logistic regression model performance and nomogram. **(A)** Forest plot of logistic regression model. **(B)** Nomogram of logistic regression model.

The logistic regression model was established using the training set with TPO-Ab, TG-Ab, 25-(OH) D, Hcy and FA. The results obtained by the established model are given in Figure 4A, and the nomogram is given in Figure 4B.

**Figure 5:**
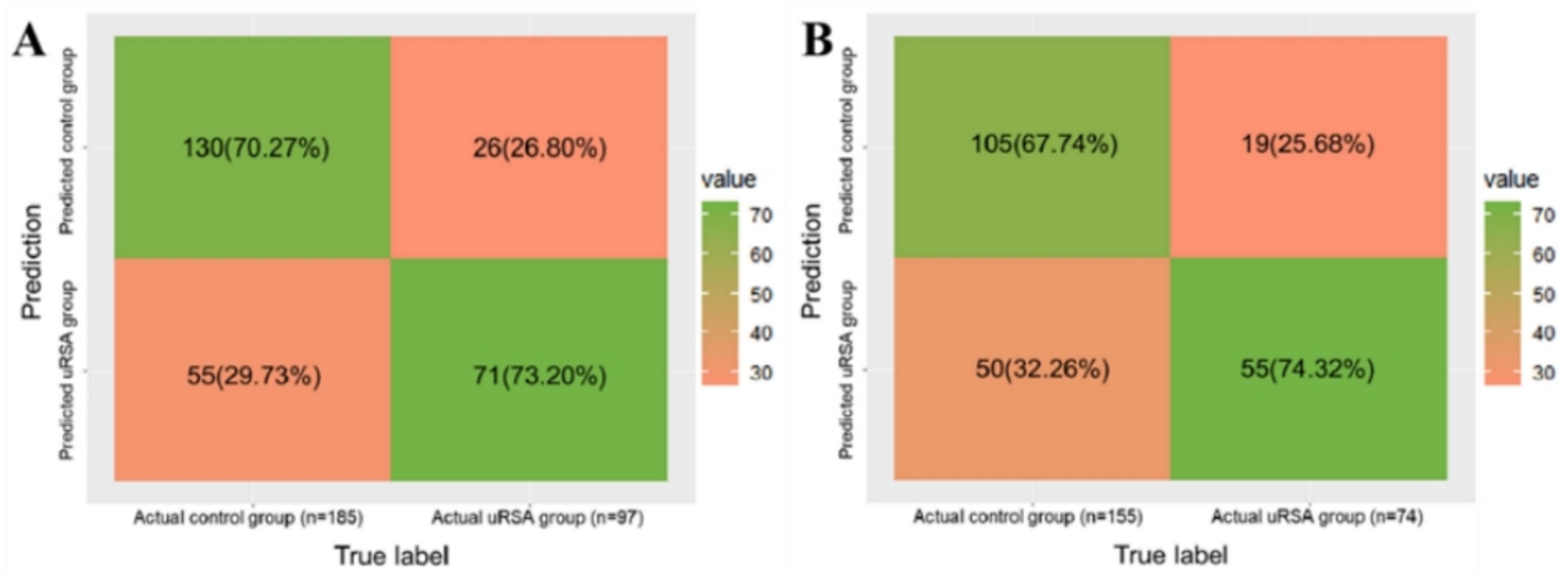
The confusion matrix of model classification result. **(A)** Testing set identification accuracy. **(B)** External validation identification accuracy.

**Figure 6:**
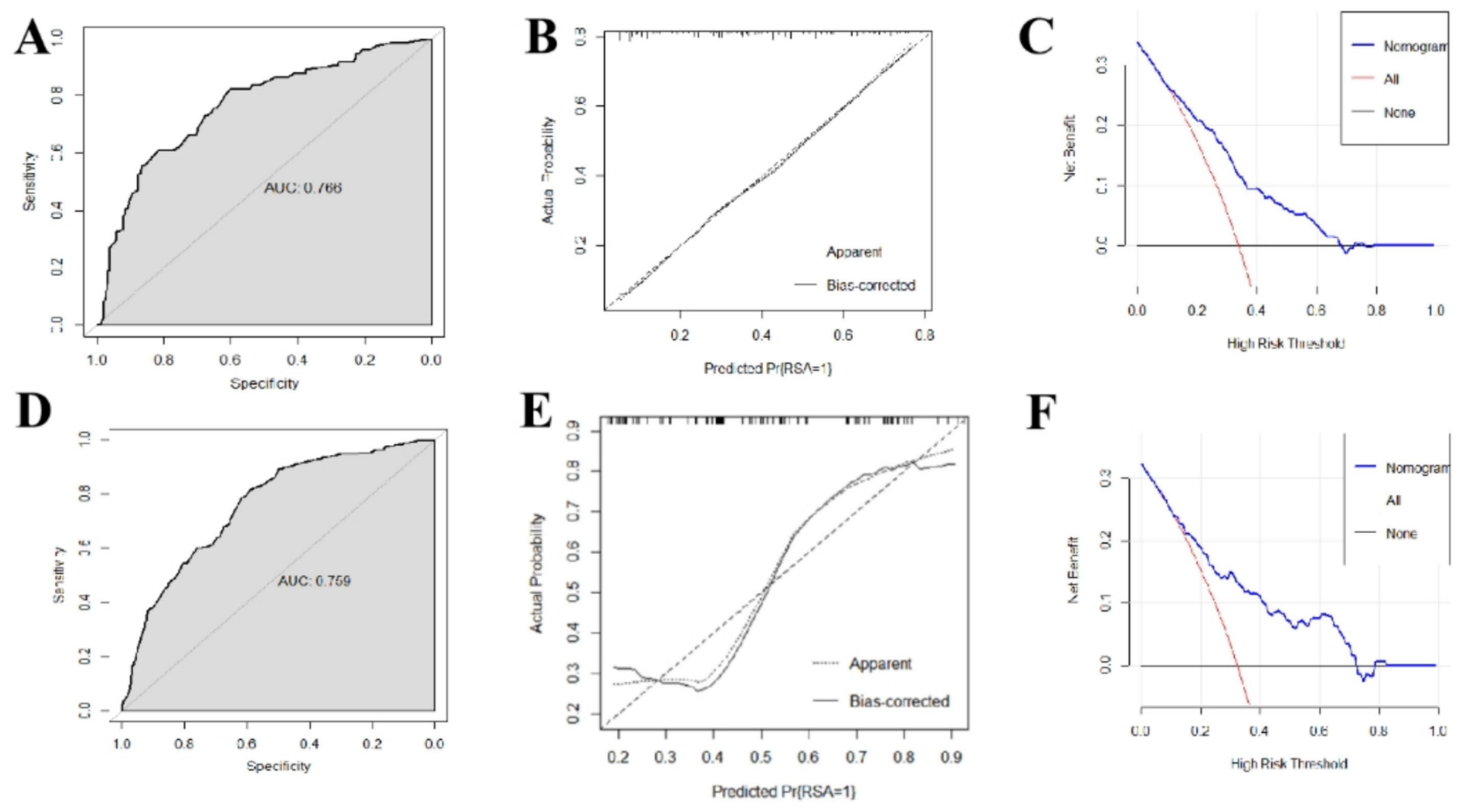
Predictive performance and clinical application value of recurrent miscarriage risk model. **(A)** ROC curve of testing set. **(B)** Calibration curve of testing set. **(C)** DCA decision curve of testing set. **(D)** ROC curve of external validation set. **(E)** Calibration curve of external validation set. **(F)** DCA decision curve of external validation set.

The performance of the model was validated using the testing set firstly. As shown in Figure 5A, the accuracy, sensitivity, and specificity of the test set were 71.28, 70.27 and 73.20%, respectively. The ROC curve was shown in Figure 6A and the area under curve (AUC) was 0.766 (95% CI, 0.678–0.854). The calibration curve shown in Figure 6B revealed good predictive accuracy between the actual probability and predicted probability.

We used the inspections of 229 women in the first trimester of pregnancy from Shangqiu Maternal and Child Health Hospital to externally validate the model. As shown in Figure 5B, the accuracy, sensitivity, and specificity of the test set were 69.87, 67.74 and 74.32%, respectively. The ROC curve is shown in Figure 6D and the AUC was 0.759 (95% CI, 0.670–0.848). Although the results of external validation were slightly worse than those of internal validation, the confusion matrix, ROC curve, and calibration curve (shown in Figure 6E) still indicated that the model has good universality.

Figures 6C,F showed the DCA decision curves of testing set and external validation set using the model proposed in this study. When the threshold probability is between 0.35 and 0.65, intervening on patients on the basis of the prediction model led to higher benefit than the alternative strategies of intervening on all patients or intervening on no patients both on testing set and external validation set. Using this model to determine whether a patient was at high risk for RPL would improve clinical outcomes.

## Discussion

This was a retrospective study between January 2019 and December 2023. Characteristics of 805 participants from Henan Provincial People’s Hospital was used for model training and internal testing, and characteristics of 229 participants from Shangqiu Maternal and Child Health Hospital was used for external validation. In this study, we investigated the relationship between routine inspections in the first trimester of pregnancy and RPL, and tried to predict the risk of RPL before the first miscarriage. According to the results of chi-square test, the logistic regression model was established using the training set with TPO-Ab, TG-Ab, 25-(OH) D, Hcy and FA. The confusion matrix, ROC curve, and calibration curve of internal testing set and external validation set showed that the model proposed had acceptable accuracy and universality in the risk prediction of RPL. The DCA decision curves indicated that the proposed model had clinical significance. Considering that the inspection items were collected before the first pregnancy loss and some RPL causes such as chromosomal abnormalities may not be clearly reflect in the detection results, we believe that the accuracy of the proposed model is acceptable and has clinical guidance significance. There is no significant deterioration in the results of external verification, whicDh confirmed the generalizability of the study results.

Endocrine function is crucial to the establishment and maintenance of pregnancy (5, 11, 16, 17). Thyroid abnormalities associated with RPL include hypothyroidism, hyperthyroidism, and thyroid autoantibody abnormalities (5, 18). TGAb and TPOAb are both thyroid specific antibodies. Clinical studies have found that abnormally high level of TPOAb can lead to the occurrence of autoimmune diseases, which in turn produce autoimmune effects on fetal cells and cause miscarriage (16, 18). Recent studies have shown that women with positive TPOAb are at higher risk of recurrent miscarriage (16, 18). Abnormal elevation of TGAb level indicates abnormal thyroid follicular structure in patients, leading to increased responsiveness to the placenta and affecting the quality of the placenta and embryo, thereby increasing the risk of miscarriage (5). In this study the TG-Ab level and TPOAb level in the RPL group were both higher than those in the control group and the difference was statistically significant, consistent with relevant research conclusions.

Vitamin D is a kind of vitamin synthesized by ultraviolet light or a small amount of food, 25 hydroxyvitamin D is the main circulating form of vitamin D in the blood, good stability, is recognized as a reliable indicator to evaluate the nutritional status of human vitamin D (10, 19–22). Its role in immune function, healthy cell division, and bone health has been supported by extensive research, and many studies have found that low serum vitamin D levels are not only associated with certain types of cancer, autoimmune diseases, but also with the occurrence of spontaneous abortion (23). Some studies have found that vitamin D may be involved in the genetic polymorphism of CYP2R1, leading to the occurrence of recurrent abortion. The AG and GG genotypes of rsl2794714 of CYP2R1 increase the risk of RPL, while the AG genotype of rsl2794714 of CYP2R1 is closely related to vitamin D levels (24). In a cross-sectional study, plasma levels of 25 hydroxyvitamin D and 25 hydroxyvitamin D-1α hydroxylase were measured in early pregnancy (7 to 9 weeks) of 120 women of childbearing age who were not pregnant, active pregnancy, and spontaneous abortion (8). The results suggested that decreased serum vitamin D levels may increase the risk of spontaneous abortion. Studies have shown that in mouse models that do not express vitamin D receptors, platelet aggregation is increased, antithrombin gene and thrombomodulin are decreased, and tissue factors are increased, while supplementation with 25 (OH) D analogs can regulate the expression of thrombomodulin and tissue factors in monocytes to improve coagulation status which can reduce the risk of RPL (25). Some studies have found that the expression of FOXP3 gene in peripheral blood of patients with recurrent abortion is significantly decreased, and the expression of FOXP3 is increased after supplementing vitamin D (21, 26). Studies have shown that vitamin D is involved in follicle development and steroid hormone production, promotes follicle maturation and ovulation by inhibiting the expression of AMH receptor and follicle-stimulating hormone receptor, affects embryo quality, promotes granule cell luteinization by inducing the reaction of key steroid enzymes, such as 3β hydroxysteroid dehydrogenase, and maintains progesterone levels to prevent miscarriage (10, 20). In summary, the etiological mechanism of vitamin D and recurrent abortion is directly or indirectly related to genetics, pre-thrombotic state, immunity and other aspects, which is also consistent with the conclusion of this study.

Serum homocysteine is a sulfur-containing amino acid present in plasma and an intermediate product of the metabolism of methionine and cysteine (27, 28). As a metabolite to maintain the normal operation of the human body, the serum homocysteine concentration tends to decline during normal pregnancy (27). In early pregnancy, high levels of homocysteine can significantly inhibit the formation of villous vessels, thus reducing the amount of blood supply to the embryo and leading to embryo death (27). Folic acid is an essential substance in Hcy metabolic pathway. Folic acid deficiency is one of the important reasons leading to abnormal Hcy metabolism and hyperhomocysteinemia. Relevant studies have shown that the serum Hcy level of non-pregnant RPL patients are higher than that of healthy women with normal reproductive history, and the folic acid level is lower than that of healthy women with normal reproductive history (11). A retrospective cohort study in Japan found that folic acid supplementation and reduction of homocysteine levels in women with assisted reproductive technology were effective in improving pregnancy outcomes (9). In this study, the FA level of the RPL group was lower than that of the control group and the difference was statistically significant, consistent with the conclusions of relevant studies.

Machine learning methods have been widely used in the fields of obstetrics and gynecology and epidemiology, including exploring biomarkers for disease diagnosis, analyzing the correlation between test indicators and diseases, predicting the prognosis of patients, and so on (12–15, 29). A study tried to investigate the biomarkers and molecular mechanisms associated with RPL using ANN model and identified three hub genes, WBP11, ACTR2, and NCSTN (15). This study contributed to understanding the molecular mechanisms and treatment strategies of RPL, but was not helpful for predicting RPL before it occurs. A study used XGBoost algorithm to establish a predictive model for natural miscarriage after *in vitro* fertilization and embryo transfer, which featured female age, ovarian structural abnormalities, lactation, anti-Mullerian hormone, activated partial thromboplastin time, anticardiolipin antibodies, and thyroid peroxidase antibodies. The features included in this model are not routine test items, and its clinical application value is limited for women of childbearing age who naturally conceive (15). A study used clinical information, vitamin D and thyroid function measurements to establish a framework for conducting an effective analysis for RPL (29). Although the model adopted innovative machine learning algorithms, the clinical data involved in this study was limited and there was no clear indicator collection time, so the predictive value of the constructed model for RPL is limited.

Current research either delves into the molecular mechanisms underlying the pathogenesis of recurrent pregnancy loss (RPL), such as signaling pathways associated with lncRNA PART1, circRNA, and CYR61, or establishes models to analyze the prognosis of RPL patients (30–33). Unlike other studies, this study aims to provide early intervention for potential patients before RPL occurs. The primary contribution of this paper lies in the establishment of a multi-parameter combined prediction model, which can predict the risk of RPL using only routine first-trimester pregnancy examinations, making it convenient for clinical application and implementation. This model allows for the screening of high-risk groups for RPL prior to the occurrence of the first miscarriage, facilitating early clinical intervention. By applying this model, clinical physicians can assess the risk of RPL in women undergoing pre-pregnancy examinations, providing a reference for further personalized examinations and early interventions.

## Limitations of the study

Due to factors such as the data collection cycle and sample size, this study still has some limitations in data analysis and model construction. Retrospective studies inherently carry the risk of potential inaccuracies in data collection due to the reliance on existing records. To mitigate this concern, we took several measures to ensure the quality and accuracy of the information utilized in our analysis. These included rigorous data validation procedures, cross-verification with multiple data sources when available, and the exclusion of any cases with incomplete or ambiguous data. Despite these efforts, we recognize that the retrospective design may still introduce some degree of uncertainty. In future studies, we plan to address this limitation by conducting prospective research, which would allow for more controlled data collection and potentially yield more definitive results.

## Data Availability

The original contributions presented in the study are included in the article/supplementary material, further inquiries can be directed to the corresponding authors.
